# Abstracts from German Journals

**Published:** 1873-01

**Authors:** Ch. Rauschenberg

**Affiliations:** Atlanta, Ga.


### ABSTRACTS FROM GERMAN JOURNALS.

BY CH. RAUSCHENBERG, M.D., ATLANTA, GA.

*THE GENESIS OF ACUTE AND CHRONIC INFLAMMATION. By Dr. S. Samuel, at Koenigsberg.* (*Virchow's Archiv, Vol. lv, p.* 380.)

The old conception of the nature of inflammation as a disturbance of the nutrition of a part caused by increased circulation and connected with hyperæmia and heat, has been shaken to its very foundation by the experiment of Claude Bernard, who, by section of the sympathetic nerve, succeeded in establishing high graded arterial hyperæmia, with an elevation of the temperature, which, after a considerable duration, was not followed by any exudation whatever. Thus it became doubtful whether peculiar disturbances of circulation (hyperæmia) constitute the principal generating factor of inflammation, or are an essential link in the inflammatory process, and the attention of investigators was called to the probable participation of the cellular elements in the production of that pathological condition.

Pathological experiment has since succeeded in demonstrating the fact *that inflammation in all its forms is primarily based upon a local alteration of the blood itself*, amounting, from a mere separation of the red corpuscles from the colorless constituents, successively, to entire coagulation, or still further on, to local detritus of the blood, with visible discoloration and decay of its red corpuscles, thus forming an ascending scale of deep local blood lesions as the essence of the entire process.

Convinced that the question of the nature of inflammation can only be solved, not by purely anatomical investigation, but only by a course of systematic pathological investigation, and that only in this manner the basis for a rational therapy of this process can gradually be attained, Dr. S. Samuel instituted a series of experiments with a view to demonstrate the circulatory disturbances peculiar to inflammation, if such existed, and chose for his study of this process the ear of the albino rabbit, the same part where already the process of active hyperæmia had been studied and demonstrated with such unsurpassed clearness, and which, by the identity of its inflammatory phenomena with those in the human body, and by the perspicuity of its morbid changes is highly suitable for that purpose. He applied, by subcutaneous injection of fluids, and by the introduction of solids into artificially made subcutaneous pouches, various substances intended to act as irritants, with a view of producing and studying all forms and grades of inflammatory reaction at one and the same locality, in order to gain a basis for the clear understanding of that process as it is produced in different tissues and organs, not by artificial agents as in his experiments, but by the different irritants which the organism produces within itself, or to which it is frequently exposed.

In relation to the effect of these different substances he made the following observations:

*Distilled Water*, of a temperature not over 40° C. (104.0° F.) is absorbed in large quantities by the subcutaneous capillaries under very indifferent vascular phenomena. *Pure atmospheric air* itself also is not an irritant to the subcutaneous capillaries. It enters the blood-vessels, remains there in separated vesicles, at fixed places for some time. If larger quantities are injected, the blood is driven off and the air occupies, for several hours, in places, the cavity of the vessels instead of the blood, and occasions a stagnation of the same during that length of time, which, however, is soon resolved by absorption of the air, generally without any morbid consequences, unless the absorption is delayed long enough to produce desiccation of the vascular walls, by which then an inflammatory process is called into existence.

*Pure Olive Oil* is also an indifferent substance. It produces very inconsiderable hyperæmia and slight turbidity of the ear, which disappears in a few days, without any morbid consequences. The same is true of the decoctions of meat—beef, chicken essence, etc. They are absorbed and enter the circulation without producing any morbid alteration of the blood or blood-vessels.

Another group of fluids, when subcutaneously injected, produces a plain constitutional and also a well defined local effect. Three drops of oil of mustard, for instance, cause, before any local effect becomes apparent, salivation, inability to stand, spasms of different muscles, trismus, opisthotonos, etc., and very frequently death; and if the animal lives, within a few hours vascular inflammation, extensive diffuse hyperæmia and considerable tumefaction of the entire ear—a striking illustration of the erroneousness of the opinion that severe local irritants can not be absorbed by the blood.

Fluid substances of greater consistency—for instance, acetic, lactic, butyric, valerianic, and the strongest of all, formic acid—exercise similar effects, differing from each other only in degree of intensity. As soon as they are injected, the place of injection turns pale, the venous net of vessels disappears, the arterial circulation remains still undisturbed, but next day the blood in the arteries is found coagulated. The entire surface at the affected spot rises into a large bulla, which dries up and turns to a black crust, which detaches itself gradually, by pieces.

The different kinds of *alcohol and ether, chloroform and hydrate of chloral* included, produce the same form of inflammation, only less intense, while the mineral acids cause the same phenomena in less time and in a higher degree than the organic acids.

The striking effect of two substances is mentioned by the author more prominently on account of its great pathological and therapeutical interest: they are the *liq. ferri sesqui-chlorati and the liq. ammonii caustici.* A few drops of a dilution of the former (two to one aqua) causes coagulation of the blood in the arteries immediately, and very soon complete mummification of the entire vascular net, which exhibits a decided disposition to spread, and terminates in a brown incrustation of the entire locality.

The liq. ammonii caust. produces the same coagulation and mummification, terminating, however, into the formation of a green scurf, without odor or tumefaction, but a well-defined line of demarcation.

The effect of this second group of fluids, just spoken of, is characterized by great intensity, but comparatively limited extension of the local effect.

Another form of inflammation, which exhibits in the highest degree *the character of acute inflammation*, is that produced by *the volatile ethereal oils.* The local effect of these oils is like that of the oil of mustard, already spoken of, on account of the intense constitutional disturbances which it produces. The entire inflammatory process produced by these substances, occupies a period of about eight days before the extensive swelling of the ear ceases and the large bulla begins to terminate into a dry scurf. Oleum menthæ piper, cinnamomi, pini, terebinthinæ, belong to this class, and their extensive local as well as rapid constitutional effect, must undoubtedly be ascribed to *their high degree of fluidity and volatility.*

The rapid, progressive character of the inflammation produced by the oil of turpentine deserves special notice. The tumefaction caused by it extends, one day after the injection, already to the sub-maxillary region and the neck of the test-animal, and is connected with severe infiltration of the subcutaneous tissue and of the glands. The ear hangs down by the side of the head, and the animal is unable to erect it. The tumefaction often reaches the thorax, and the animal frequently perishes.

The effect of the croton oil also, somewhat different in detail, belongs to this class of inflammations.

Another class of inflammatory action is produced by more or less concentrated solutions of saline substances—for instance, *chloride of sodium, sulphate of magnesia, etc.* They produce, according to their degree of concentration, more or less intense blood-coagulation in the veins and arteries, vesicular exudation of short duration, proliferation of the vessels *with complete resolution and restitution to the normal condition.*

Aqua croci, curcumæ, produce very similar phenomena, but of much shorter duration. As the particles of coloring matter, which have caused a temporary obstruction of the veins, are removed by ensuing congestion and proliferation, resorption takes place, and these and the inflammatory phenomena following the injection of saline solutions may be considered as inflammatory resorptions or inflammations tending to resorption.

The inflammation produced by a solution of carmine, while it is exactly the same as the one just described, shows generally, after complete resolution, a retention of carmine molecules at different points of the subcutaneous tissue for weeks and months, without any further vascular or nutritive disturbances.

So far we have only considered the effect of fluid and volatile substances as instigators of inflammatory reaction. We have found that all are readily taken up by the blood-vessels of the locality of application, and that the differences in their effect depend upon the extent and intensity of the disturbance which they produce. The former is determined by their greater or smaller fluidity and volatility—a mere physical quality—the latter by their physical and chemical capacity, to produce stagnation, coagulation, or decomposition of the blood. This constitutes the main essence of the entire process, to which, however, in the milder effects of resorbed substances, dilatation and proliferation as secondary, and the latter as the only doubtless active vascular symptom, become occasionally attached.

These effects of volatile and fluid substances require a comparison with the effects of dry molecules and solid substances.

*Arsenic*, when introduced into a subcutaneous pouch of the rabbit ear, causes, in the course of one day, a white discoloration of the place of application, surrounded with a small interior red and a larger exterior bluish areola. Vesicular exudation, uniform redness and swelling around the white center, and finally a purulent margin surrounding the entire spot, with a small vascular ring, follow and terminate slowly into an incrustation of the entire affected spot, without any symptoms of constitutional poisoning or any vascular phenomena beyond the immediate neighborhood of the point of application.

*Corrosive sublimate, cantharides and tart. emeticus* produce the same inflammatory phenomena—each one, however, to a more limited extent.

*Chloride of sodium* in substance causes the same appearances as those produced by a concentrated solution of the same, only much slower and to a much more limited extent.

*Flores sulphuris* produce increased vascularity around the walls of the pouch, with a slight secretion of pus, while vermilion causes circumscribed purulent hearths.

This scale of different effects is so clear and comprehensible that a detailed interpretation is hardly necessary. The ear of the rabbit, being comparatively dry and destitute of fluids, supplies a small amount of fluid material for the solution of the resorbed substances; hence, the non-occurrence of symptoms of general intoxication and the insignificance and marked limitation of the local phenomena. Other more succulent tissues would show a somewhat increased and more extensive effect; but the fact that the solidity and the dry aggregate condition of the substances applied occasions this limited and mild effect is nevertheless sufficiently clear.

The same slow and limited inflammatory reaction follows mechanical injuries caused, for instance, by the insertion of pins. An inconsiderable exudative turbidity within twenty-four hours, and a reddish discoloration within seventy-two hours, with vascular proliferation immediately around the point of insertion, are the only phenomena following it, even if the pins are allowed to remain for months.

In consideration of what has already been demonstrated, we can not be astonished at the minuteness of the inflammatory effect of these mechanical injuries, or foreign bodies, but ought from our stand-point rather to inquire why they should cause any inflammation at all. We have seen that carmine molecules can remain within the blood-vessels any length of time without producing it, and the same is true of the pigment of the yolk of the egg. Therefore, the foreign body within itself does not produce the inflammatory phenomena, but only the mechanical or chemical alteration which it produces within the tissues. Whenever it exercises either, a qualitative change will take place in the fluids circulating within the tissue, which return, by the laws of resorption, into the blood. Carmine and yolk pigment do not interfere with the integrity of the cells, and a needle necessarily exercises, by its pressure, a slight mechanical effect; but only *a qualitative alteration of the fluids* engaged in endosmosis and exosmosis, mechanical and chemical exchange of substance within the tissues constituting the process of nutrition (which fluids issue from and return to the blood, and therefore influence its composition and quality), can constitute *a primary cause of inflammation.** The inflammation itself begins primarily with the local alteration in the blood, and this blood alteration constitutes the nucleus of the inflammatory process.

* It is perhaps not improper to remind the reader, at this point, of the probability of the existence of so-called trophic or nutritive nerves, which, like the vaso-motor nerves, are fibres of the sympathetic system, and seem not only to preside over the nutrition of the parts, in the strictest sense of the word, but also over the regulation of secretion and resorption. Physiological experiments made by Ludwig, von Wittich, and Goltz, have demonstrated the existence of such a system of nerves almost beyond a doubt; and Pflüger, Recklinghausen, Lippman, Hensen, and Eberth, have microscopically shown that nerve-fibres

With this view of inflammation, the occurrence of primary changes in the parenchyma is not denied; its position in the order of the phenomena which constitute the inflammatory process is only changed.

All well-established facts contained in the above-mentioned observations show only passive alterations of the parenchymal cells, caused either by temporary inundations with foreign fluids, which were resorbed without visible changes (solutions of saline substances), or by destruction and chemical alterations (organic acids, ethereal oils, etc.), or by mechanical disturbances (pressure, contusion by needles, etc.); and finally, no alterations of any kind were observed when carmine or yolk pigment was deposited and remained within the tissues.

The circulatory disturbances of the inflammatory process undoubtedly occur in the middle, between the passive and active cell alterations and the proliferation of vessels, and still more so that of cells; the active cell changes are by no means primary, but secondary phenomena in this process, and all those inflammations which terminate in resolution run their course without any active cell changes in the parenchyma.

In conclusion, Dr. S. Samuel condenses the results of his experiments in the following theses:

1. *The nucleus of the entire inflammatory process is always a local blood change*, which extends from mere stagnation to complete decomposition, coagulation and decay within the affected vessels.

2. Fluid and volatile substances, which are capable of occupying rapidly a large complex of vessels, cause the most pregnant forms of acute inflammation.

3. Solid and dry substances cause, in the non-succulent tissue of the rabbit ear, chronic inflammations in proportion to their diffusibility.

4. Thus the aggregate condition of the substances causing inflammation presents a graduated measure of their effects, the enter the cells of the lymphatic glands, of the salivary glands, of the liver, the pancreas, and the cells of other tissues.

If these nerves answer the purpose ascribed to them, the conclusion that morbid disturbances of their function would affect the organic chemical process, the metamorphosis of substance upon which the integrity of a part depends, and would give rise to local blood changes (the primary cause of inflammation alluded to by our author), is quite rational and near at hand.—CH. RAUSCHENBERG.

extreme poles of which are represented on the one hand by that of needles and indifferent pulverized substances, and on the other by that of ethereal oils.

5. The intensity of the process depends upon the mechanical and chemical changes produced by the resorbed substance causing inflammation. The severe forms of blood coagulation are insoluble; the lighter ones, up to a separation of the blood-constituents, can terminate in resolution. Concentrated organic acids furnish examples of the first, and diluted saline solutions examples of the latter form. Dilatation and proliferation of the vessels can only occur where blood-coagulation has not already taken place.

6. As primary effects of foreign bodies in the parenchyma, have to be considered: (*a*) Destruction and chemical alteration of the cells; (*b*) inundations with indifferent fluids, which are afterward resorbed; (*c*) mechanical injuries by pressure, contusion, etc.; (*d*) foreign bodies remain for months without any morbid effects—for instance, carmine. All the primary changes of the tissue are of a passive character. The active tissue-changes occur at a later period of the process.

7. Soft, tenacious, and particularly mucilaginous substances, which can not bo resorbed in toto, do not interfere with the maintainance of a continuous blood-current, and do not produce coagulation, but are the most efficient instigators of *chronic suppuration.*

8. Causes of inflammation which, when absorbed, do not produce insoluble coagulation, enter the general circulation directly or indirectly, and produce the constitutional effect either sooner than the local one (for instance, oil of mustard), or later by the resolution of the stagnations (for instance, in the so-called inflammatory resolutions.)

9. Oil of turpentine represents the most marked example of acute progressive inflammation, and is best suited for the study of the temperature questions connected with the inflammatory process, while the effect of petroleum is best adapted to the study of the process of suppuration.

10. Indifferent substances are only those which have no capacity of interfering with the normal condition of the intermediate lymph-current in the tissues, or of producing at once, or gradually, local alterations of the blood.

### *THE TRANSFUSION OF BLOOD.*

One of the most interesting articles contained in the surgical part of Volkmann's "Collection of Clinical Lectures,"* now in course of publication in Germany, is that of Dr. H. Leisrink, on the transfusion of blood.

He represents this operation as one which, not only on account of its preëminently life-saving effect, the simplicity of its execution, and its almost entire freedom from danger, should become the common property of all physicians, but which also, on account of its strictly physiological and rational basis, is destined to occupy, in the future, one of the first and most brilliant positions as a surgical remedy in the practice of medicine. Here follows a short recapitulation of the history of the operation. It was first practiced on the dog in 1666, by Lower, and successfully on men in 1667, by the same one, and by King, in England, and by Dennis, in Paris. It was afterward, it seems, entirely forgotten, as it does not appear on record for over a century; but with the dawn of modern physiology and improved surgical instruments, it was resurrected by James Blundell, in 1824, who performed four successful transfusions. Soon afterward Dumas, Provost and Dieffenbach became its warm advocates. Martin† recommended it in the hemorrhages connected with the parturient state, Kühne‡ in carbonic-oxyd poisoning, Neudörfer in chronic anæmia, Nusbaum§ in chlorosis.

The celebrated Hueter advocated in two classical essays the transfusion of blood into the arteries.‖

The most important practical points connected with this operation are contained in Dr. Leisrink's answers to the following three questions:

1. What are the indications for transfusion at the present state of surgical science?
2. Does it require defibrinized or non-defibrinized blood?
3. What is the best and simplest method of performing it?

* Sammlung Klinischer Vorträge, von Richard Volkmann, No. 41, July, 1872.

† Uber die Transfusion Neuentbundenen, Berlin, 1859.

‡ Virchow's Archiv, xxvii, 1863.

§ Centralblatt, No. 9.

‖ Von Langenbeck's Archiv, Vol. xii; Centralblatt, 1869, No. 25.

First. *Transfusion is indicated in all morbid conditions where the blood, quantitatively or qualitatively, is so altered that it can not perform its physiological duties.*

*Acute anæmia* following severe losses of blood in operations, in traumatic injuries, in parturient or recently delivered females, was until quite lately the only condition where transfusion was practiced, and where—as it is only the quantity of the blood which in these cases of otherwise generally healthy individuals has to be elevated to the normal standard—transfusion is generally followed by the most gratifying results. The hemorrhages of tuberculosis, dysentery and similar diseases are necessarily excluded from this category, as increased blood-pressure would not be desirable in such cases.

*Excessive hemorrhages* connected with or following traumatic injuries, operations, etc., where the injured parties under the the common regimen make often tedious recoveries, and suffer sometimes for years from the debilitating influences of the loss of blood which they have sustained, require the undelayed execution of transfusion. Prompted by the correctness of this thesis, transfusion has become an operation of almost daily occurence in the large surgical clinics of Germany. Esmarsch has in the extirpation of a fibro-cavernous tumor from the base of the skull, and also in an exarticulation of the femur at the hip-joint, directly counteracted the great loss of blood by transfusion, respectively into a vein of the arm and the femoral vein, of the very blood which was lost during these operations. Volkmann has done the same in three cases of exarticulation of the hip-joint, and has first recommended this course.* Thus transfusion has become a valuable and efficient auxiliary in the successful execution of severe surgical operations, and will be continued to be used in this direction with increasing success.

The hemorrhages occurring within and immediately following the parturient state require special notice. *Transfusion must not be delayed where the capacity of the womb to contract after the expulsion of the fœtus seems temporarily suspended*, and where the amount of blood lost and the condition of the pulse and other symptoms indicate immediate danger. In all doubtful cases transfusion must be performed anyhow, as it is—when properly

* Uberdrei Fälle von Exarticulation des Oberschenkels im Hüftgelenke. Deutsche Klinik, 1878, No. 42 and 43.

and carefully performed—an absolutely harmless operation. At the same time, it is important to bear in mind what Martin has undeniably demonstrated—*that injection of blood into a vein is the most prompt and most efficient irritant of a relaxed and inactive womb.*

The use of transfusion in cases of chronic anæmia, brough on gradually by deficient nutrition or other internal causes, has been less readily accepted, although the conditions indicating it are approachingly the same as those existing in suddenly produced anæmia. Patients suffering from an incurable disease, which continually reduces the quantity and quality of the blood, would not be fit subjects for transfusion; but cases in which, after long continued febrile diseases—for instance, typhus or typhoid fever—convalescence continues to go on very slowly and imperfectly, and where an intercurrent disease might easily extinguish the flickering light of life, will occasionally offer very pointed indications for the use of this remedy.

Evers and Jürgensen have lately published a number of such cases, among which striking improvements and cures by the use of transfusion were recorded. Cases of chronic gastric catarrh particularly belong to this class. An injurious circulation and deficient quantity and bad quality of the circulating fluid prevents the secretion of normal digestive fluids, the formation of a sufficient quantity of good chyle, and therefore of healthy blood; hence the cachectic state of the system in such cases. Transfusion of good blood improves the secretion of gastric juice, good hydro-chloric acid pepsin is furnished, digestion becomes normal, and convalescence is ushered in. In these cases the heart has been accustomed to a small supply of poor blood; a large blood-wave reaching it at once would overtask its propulsive powers; hence *small but repeated transfusions*, after a small quantity of blood has previously been allowed to flow from the vein selected for the performance of that operation, constitute the proper method of application in those cases.

Among the abnormal qualitative changes of the blood, which furnish an indication for the use of transfusion, pyæmia and septicæmia occupy the first, poisoning with carbonic oxyd, sulphuretted hydrogen and carbonetted hydrogen-gas, the second rank.

Small quantities of good blood, introduced as frequently as possible, with proper hospital hygiene and surgical treatment to arrest the formation of the microscopic fungi or animals which cause the disease, have often accomplished, if not brilliant, at least very encouraging results in the first two marked conditions.

In poisoning with deleterious gases, which generally enter into chemical combinations with the red corpuscles and deprive them of their faculty of uniting with oxygen, as carbonic oxyde does, or make them otherwise unable to answer their physiological duties, a new supply of good blood is a radical remedy. Hence venesections, as large as the nature of the individual case will safely permit, to get rid of as much poisoned blood as possible, followed immediately by proportionately larger transfusion, to supply as much good blood as possible, soon repeated if necessary, are successfully used in these cases. In poisoning with opium, atropine, chloroform, etc., the transfusion deserves a trial upon the same principle.

An abnormal histological condition of the blood, such as exists in chlorosis and leucæmia, furnishes another indication for the practice of transfusion. Nusbaum has used it in chlorosis; Mosler in leucæmia, but with less permanent effect.

Second. *Does transfusion require defibrinized or non-defibrinized blood?*

The process of defibrination of the blood requires but a few minutes; hence a very immaterial delay in the execution of the operation, the effects of which cannot be compared with the undeniable dangers of embolism and thrombosis attached to the use of non-defibrinized blood. Hence, *only defibrinized blood must be used for tranfusion*, even if the great benefit of the arterialization, which the venous blood undergoes while being stirred, were not taken into consideration.

The venous blood is defibrinized by stirring it with a small stick of wood for a few minutes; then it is filtered through a fine piece of clean linen, and then stirred until no more fibrin attaches itself to the stick, and then filtered again. During this procedure the vessel containing the blood is kept standing in another one filled with water of about 38° C. (100.4° F.) temperature. One or two degrees more or less make no difference, a fact worthy of consideration, as themometers, particularly in the country, are not always at hand.

*How is the operation performed?*

A sharp knife and a good syringe would in case of unavoidable necessity be sufficient for the performance of the same; but instruments expressly fitted up for its execution make it necessarily easier and simpler.

Two kinds of syringes—one constructed by Eulenburg, the other by Uterhardt—are now principally used in Germany. The latter is preferable on account of the great simplicity of the contrivance by which any air contained within its cavity is prevented from being drawn forward with the blood.

Besides the syringe, nothing is needed but a pincette, a bistoury, a needle, and a few pieces of thread.

In all cases where a qualitative change of the blood exists, a venesection a little larger in quantity than the amount of blood to be injected must immediately proceed the transfusion. It is very difficult to give an average measure for the amount of blood to be injected, as different considerations contained in the nature of the disease and the constitution of the patient must in each case determine what amount will most probably create the most beneficial effect. Hueter thinks that from seven to eight ounces would answer best in a large number of cases, but smaller and larger quantities have been used in the different cases on record.

Where a large loss of blood has simply to be resupplied, or blood poisoned by noxious gases as much as possible diluted, it is essential to introduce *as large a quantity of blood at once as can be done safely*, and less than 250 grammes, (about 16 ounces,) are hardly ever used; but the operation is soon repeated if necessary.

Where a heart weakened by a dissolute condition of the blood—for instance in pyæmia and septicæmia, or by chronic cachexy in chronic anæmia, chlorosis, leucæmia—would be unable to propel a much larger quantity of blood than it has been accustomed to during the existence of the disease, *smaller transfusions*, 100 to 150 grammes (about six to eight ounces), *frequently and perseveringly repeated*, are the secret of success.

A healthy and stout man must be at command to obtain the required amount of blood by venesection. The blood is caught in a clean vessel, and defibrinized in the above described manner.

When ready for use, it is kept warm by remaining under the influence of the heated water until it is needed.

A conveniently situated surface vein—for instance, at the bend of the arm—is selected, exposed by a longitudinal incision and carefully separated from the surrounding tissues. Now the vessel is ligated below, to keep the blood away from the field of operation. A ligature thread is passed under the vein at the upper edge of the incision, but not tied. The syringe is next filled with blood, and also the canula, which is introduced into the vein through an incision. The syringe is inserted into the canula, and the blood is driven into the vein by a slow rotating motion of the piston.

Some one must stand on the opposite side of the patient, with one hand on the pulse, counting its beats aloud, and the operator himself must watch the respiratory movements of the non-chloroformed patient. A slight change in the pulse is always observed: it becomes either faster or momentarily smaller. Slight momentary exertions to cough, and restlessness of the patient occur, but need not alarm the operator. Severe oppression of the chest and continued irregularities of the pulse demand caution; fainting, the interruption of the operation. Some of these troubles will certainly be avoided by quieting the fears and apprehensions of the patient in due time; The more severe and alarming ones are, however, eventualities of very rare occurrence, but had to be mentioned to do the subject justice. Where the operating physician has professional assistance, the operation can be performed in half the time by dividing the labor.

Sometimes the canula rests too firmly against the walls of the vessel, and does not allow the blood to pass; a slight retraction and adjustment of it in the direction of the longitudinal axis of the vein will amend this difficulty at once. Whenever the desired amount of blood has been injected, the canula is withdrawn, and the vein closed by tying the upper ligature. After dividing the vein between the ligature, the incision wound is closed by one bloody suture, and a few turns of a roller bandage are applied.

Dr. Leisrink, in conclusion, expresses the opinion that venous transfusion will always maintain its superiority over the operation on an artery.

The difficulty experienced in driving a sufficient quantity of blood into an artery, and the great possibility of secondary hemorrhage, are the disadvantages, the impossibility of an entrance of air the advantage, of the arterial operation. The latter, however, can be easily avoided in the venous operation by care and a good syringe. Thus the transfusion into an artery will at least never become an acceptable operation in private practice.

The object of Dr. Leisrink and others in practicing transfusion has been to establish an effective weapon by which diseases, so far incurable, can be successfully combatted, and a sincere desire to gain co-laborers in the continuation of this important work has caused the publication of the paper, the main features of which are contained in this article.

